# When Do Team Members Share the Lead? A Social Network Analysis

**DOI:** 10.3389/fpsyg.2022.866500

**Published:** 2022-04-25

**Authors:** Sebastian Tillmann, Hendrik Huettermann, Jennifer L. Sparr, Sabine Boerner

**Affiliations:** ^1^Chair of Management (esp. Strategy and Leadership), University of Konstanz, Konstanz, Germany; ^2^Chair of Leadership and Organizational Behavior, Bundeswehr University Munich, Munich, Germany; ^3^Department of Management, Technology and Economics, Chair of Work and Organizational Psychology, ETH Zürich, Zürich, Switzerland

**Keywords:** shared leadership, political skill, empowering leadership, social network analysis, leadership emergence

## Abstract

Shared leadership is not only about individual team members engaging in leadership, but also about team members adopting the complementary follower role. However, the question of what enables team members to fill in each of these roles and the corresponding influence of formal leaders have remained largely unexplored. Using a social network perspective allows us to predict both leadership and followership ties between team members based on considerations of implicit leadership and followership theories. From this social information processing perspective, we identify individual team members’ political skill and the formal leaders’ empowering leadership as important qualities that facilitate the adoption of each the leader and the follower role. Results from a social network analysis in a R&D department with 305 realized leadership ties support most of our hypotheses.

## Introduction

Many organizations are facing the challenge to successfully adapt to volatile business environments and fast-changing customer needs. As one way to increase flexibility and innovativeness, they decentralize their structures and rely on less-hierarchical forms of leadership (Lee and Edmondson, 2017). That is, many teams in contemporary organizations consist of members with high levels of expertise that solve complex problems and develop creative solutions (Mathieu et al., 2017; van Knippenberg, 2017). Given such challenging demands, these teams rarely rely on one single hierarchical leader alone who performs all necessary leadership functions. Rather, many teams also rely on shared leadership—that is, team members sharing the lead between each other to reach common goals (Pearce and Conger, 2003)—and distribute leadership responsibility based on team members’ relevant expertise (Wang et al., 2014).

Mirroring this trend toward more shared forms of leadership in organizational practice, also organizational scholars have expanded their focus beyond hierarchical approaches to leadership. As Lord et al. (2017) illustrate, shared team leadership has gained particular prominence in the third and most recent wave of leadership research over the past decades, following up on more traditional paradigms that focus on single hierarchical leaders. Thus, a considerable number of studies in recent years have examined the consequences of shared leadership in teams, with meta-analyses showing positive effects on team functioning and effectiveness (Nicolaides et al., 2014; D'Innocenzo et al., 2016). Given its positive effect on team success beyond the impact of formal leadership (Pearce and Sims, 2002; Ensley et al., 2006; Nicolaides et al., 2014), research has begun to explore the question of how shared leadership in teams can be promoted (Wassenaar and Pearce, 2018; Wu et al., 2018; Zhu et al., 2018).

However, the majority of the few existing studies has examined antecedents of shared leadership at the team level, thereby focusing on the average extent to which team members engage in the leader role. While this research is insightful, prior analyses have largely neglected the within-team processes of shared leadership that comprise both, being relied on *and* relying on others for leadership (Small and Rentsch, 2010; Chrobot-Mason et al., 2016). For example, Carson et al. (2007) show that the internal team environment enhances the average level of shared leadership in the team (i.e., team members taking the leader role) but do not explain why and how individual team members rely on one another for leadership. As leadership can only exist if there are also followers and follower behaviors (DeRue and Ashford, 2010; Uhl-Bien et al., 2014), shared leadership requires team members who are able and willing to take turns in both the leader and follower role. Thus, it is essential to understand the drivers of both team members’ reliance on others for leadership (i.e., taking the follower role) and being relied on for leadership (i.e., taking the leader role). Yet, scholars have only recently begun to study antecedents of these within-team processes constituting shared leadership. Thus far, limited insights exist on factors relating to either seeing others as leaders or being seen as a leader, for example, team members’ level of organizational identification. However, research on the question of what enables individuals to fill in each of these roles is still scarce (Chrobot-Mason et al., 2016; Klasmeier and Rowold, 2020).

Another major limitation of the current literature concerns the limited understanding of the formal leader’s role for shared leadership. This question is highly relevant since most organizations do not entirely decentralize their hierarchical systems but rather rely on a combination of formal and shared leadership; thus, shared leadership often takes place in teams that still have a formal leader (Pearce and Conger, 2003; Nicolaides et al., 2014; Lee and Edmondson, 2017). Yet, extant research has primarily focused on the direct influence of formal leadership on shared leadership at the team level (Pearce and Sims, 2000; Hoch, 2013; Klasmeier and Rowold, 2020), while only recently scholars have begun to explore more complex constellations in which formal leadership promotes and interacts with shared and emergent forms of leadership (e.g., He et al., 2020; Chiu et al., 2021; Ziegert and Dust, 2021). Still, the question of whether and how formal leadership facilitates the processes that allow team members to engage not only in the leader but also in the follower role has remained unanswered thus far.

To address these gaps in research on antecedents of shared leadership, we employ a social network perspective which allows us to focus on leader-follower ties between individual team members rather than the average extent of shared leadership at the team level. Building on the concept of implicit leadership and followership theories—which has recognized the potential difficulty of moving from one role to the other (Lord et al., 2020)—we identify political skill as a relevant social effectiveness quality that may enable individual team members to actively, flexibly, and convincingly engage in the leader and the follower role. Politically skilled individuals “understand social situations well, and can accurately interpret their behavior and the behavior of others” (Ferris et al., 2007, p. 292). As such, political skill has repeatedly been theorized as a predictor of shared leadership but has not yet been empirically examined (Ferris et al., 2009, 2012; Russell et al., 2016). We extend and test this notion by proposing that their superior understanding of situational and others’ needs enables politically skilled team members to show team-prototypical leader behaviors which other team members are willing to rely on, as well as team-prototypical follower behaviors that signal the willingness to rely on leadership from other team members.

In addition, we acknowledge the relevance of formal leadership as a supportive context for shared leadership (Nicolaides et al., 2014). In particular, empowering formal leadership was shown to be positively associated with shared leadership at the team level (Pearce et al., 2008; Hoch, 2013; Fausing et al., 2015). With our focus on within-team processes of shared leadership, we extend this earlier perspective by proposing two important functions of empowering formal leadership. First, empowering formal leaders grant power, responsibility, and discretion to team members and thus create a promotive context for shared leadership (Manz and Sims, 1987; Lee et al., 2015; Sharma and Kirkman, 2015). Second, they provide a prototypical role-model for shared leadership as empowering leadership comprises behaviors related to both engaging in the leader role and encouraging others to take over responsibility (i.e., with the formal leader taking a follower-like role). We propose that politically skilled team members take advantage of this promotive context and the formal leader’s prototypical role modeling to master the leader and the follower role in their team.

**Figure 1:**
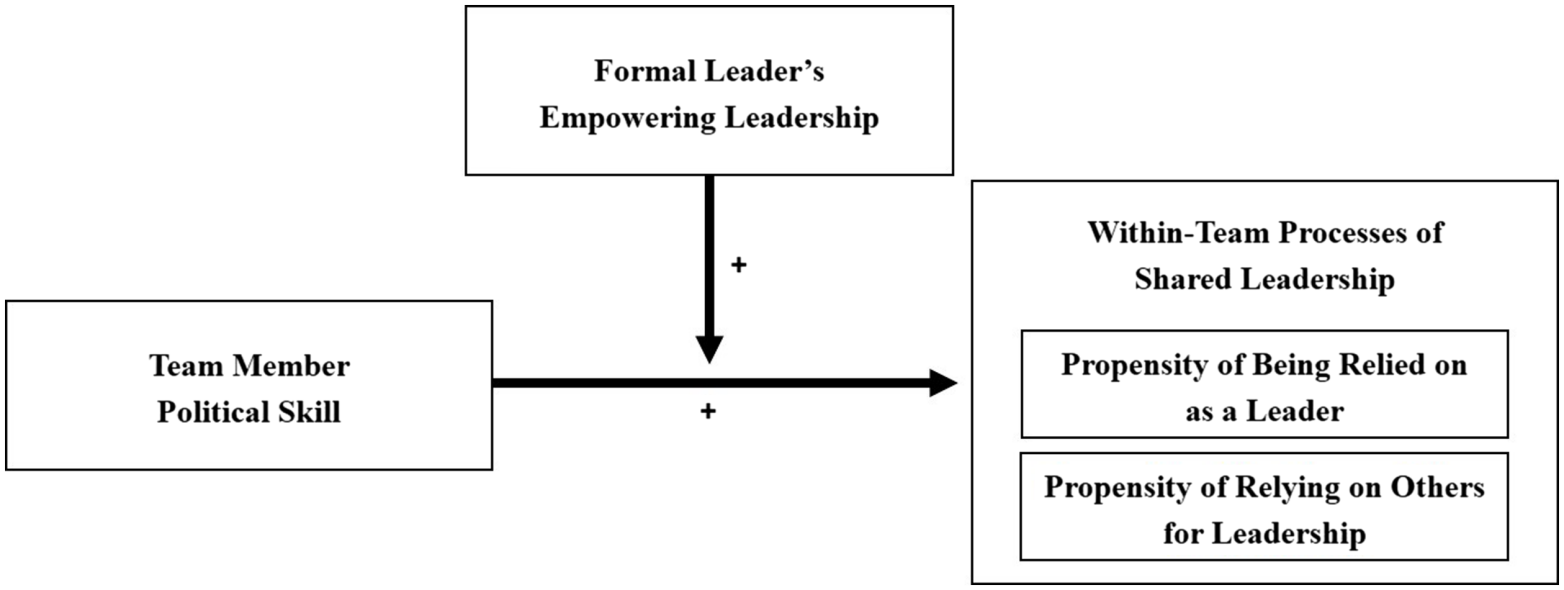
Theoretical model of the moderated influence of political skill on shared leadership.

With our focus on within-team rather than team-level dynamics of shared leadership, we place our research at the intersection between the emergent leadership literature, which explains the emergence of single informal leaders, and the shared leadership literature, which investigates the collective leadership influences within teams (Hanna et al., 2021). From this perspective, we acknowledge the importance of team members engaging in both leadership and followership roles in order to share the lead. In developing and testing our theoretical model (see Figure 1), we thus make two major contributions to the shared and emergent leadership literatures.

First, we identify and analyze political skill as a potential driver of both being relied on and relying on others for leadership. This allows for a deeper understanding of the personal qualities that help team members to emerge not only in the leader role, but also in the follower role which is required for shared leadership. Furthermore, the focus on political skill adds to the scope of the emergent leadership literature, which to date has rarely considered social skills such as political abilities (Acton et al., 2019), but rather focused on individual attributes and abilities such as self-monitoring (Dobbins et al., 1990) and cognitive ability (Rubin et al., 2002), as well as demographic factors such as gender (Badura et al., 2018), nationality (Paunova, 2015), and personality (Ensari et al., 2011). In addition, the focus of our analysis contributes to the political skill literature by adopting a network perspective (Scott et al., 2018) on the concept which opens a new line of research beyond the impact of political skill on formal leaders’ emergence and effectiveness (Kimura, 2015).

Second, we consider the interplay of individual team member qualities with the formal leadership context to explain when team members fill in each the leader and the follower role in shared leadership. By taking multiple antecedents into account, we go beyond the majority of prior research that has focused on single drivers of shared leadership. In addition, we provide a more nuanced view of the role of formal leadership beyond the direct effects considered in the majority of prior research (e.g., Hoch, 2013). Our moderation model offers a unique understanding of empowering formal leaders’ enabling role on team members’ use of their political skill to adopt each of the roles required for shared leadership, namely, for being relied on and relying on others for leadership. Thus, our findings have important practical implications for the selection and training of both members and leaders in teams with shared leadership.

## Theory

### Antecedents of Shared Leadership From the Within-Team Perspective

Shared leadership is most commonly defined as “a dynamic, interactive influence process among individuals in groups for which the objective is to lead one another to the achievement of group or organizational goals” (Pearce and Conger, 2003, p. 1). Shared leadership is concerned with the informal sharing of leadership among team members (Denis et al., 2012) and typically considers average leadership influences of multiple leaders within a team (Carson et al., 2007).

In the present study, we assume a social network perspective of shared leadership based on leadership and followership relations (i.e., leadership and followership ties) between individuals, thereby placing our research at the intersection between the individual level that is considered in the emergent leadership literature (Zhu et al., 2018; Hanna et al., 2021) and the team level that is mostly adopted in the shared leadership literature. We posit accordingly that shared leadership takes place between the team members who take turns to adopt complementary roles, namely, the leader and the follower role. Thus, we acknowledge that team members who assume a leadership role depend on other team members to accept their leadership (i.e., relying on them for leadership) by assuming the follower role (DeRue and Ashford, 2010; Chrobot-Mason et al., 2016). Since team members take turns in the leader and follower roles, we focus our research on antecedents of team members’ engagement in each of the roles.

The shared leadership literature, where scholars have traditionally focused at the team level, distinguishes between different categories of team-level antecedents, such as group, task, and environmental characteristics (e.g., Pearce and Sims, 2000; Carson et al., 2007). For example, recent empirical evidence has demonstrated the relevance of factors such as a team’s demographic and dispositional composition, team climate, and formal team leaders’ transformational leadership as drivers of shared leadership (Klasmeier and Rowold, 2020; Kukenberger and D'Innocenzo, 2020; Siangchokyoo and Klinger, 2022). Accordingly, meta-analyses have clustered the empirical evidence of shared leadership antecedents around the categories of formal team leader factors, the internal team environment, and team characteristics (Wu et al., 2018; Zhu et al., 2018). In contrast, the emergent leadership literature has focused more on individual-level antecedents of leadership emergence, such as personality (Ensari et al., 2011), gender (Badura et al., 2018), leadership-related mental models (Evans et al., 2021; Wellman et al., 2022), or the risks perceived to be associated with taking an informal leadership position (Zhang et al., 2020). However, there is also evidence that individuals’ social abilities can foster informal leadership emergence. For example, Walter et al. (2012) demonstrate the relevance of emotion recognition in others for the emergence of leaders, while Li et al. (2012) find that emotional stability predicts leader emergence. Other authors find empirical support for a positive influence of self-monitoring behaviors on leadership emergence (Dobbins et al., 1990). These earlier findings indicate the importance of characteristics and abilities that allow team members to build and maintain relationships with others, while also having a good level of control over themselves.

In our research, we focus on the network of leadership (i.e., being relied on for leadership by others) and followership (i.e., relying on one another for leadership) relationships between the team members. From the perspective of this shared leadership network, we aim to identify antecedents that predict both of these relationships. To successfully engage in the leadership and the followership role, individuals need other team members to ascribe the respective role to them and to assume the complementary role for themselves (DeRue and Ashford, 2010). We draw on research on implicit leadership and followership theories (i.e., ILTs and IFTs), which is particularly insightful when it comes to understanding these ascription processes (Scott et al., 2018; Lord et al., 2020). ILTs and IFTs are individually held cognitive frameworks that provide the structure for processing information about leadership and followership based on shared assumptions about prototypical leader/follower traits and behaviors in the team (Epitropaki and Martin, 2005; Uhl-Bien and Pillai, 2007). Thereby, individuals construct prototypical assumptions about leader and follower behaviors based on their own experiences. They also include in these assumptions their own role as either a leader or follower in the future, e.g., whether they see themselves in such roles (Epitropaki et al., 2017; Lord et al., 2020).

In the following, we specifically identify political skill as a unique set of social competencies that enable team members to appear as prototypical leaders and thus get others to rely on their leadership, as well as to appear as prototypical followers, which allows them to rely on others for leadership. Furthermore, we build the case for empowering leadership of the formal leader to build a promotive context and to provide a prototypical role-model for shared leadership (i.e., the formal leader influences team members’ implicit theories about prototypical leader and follower qualities). The resulting moderation model (see Figure 1) acknowledges the interplay between team members’ assessment of their own social skills and their perceptions of formal leadership as an important context variable for shared leadership for their engagement in each the leader and the follower role (Wellman et al., 2019; Hanna et al., 2021).

### Individuals’ Political Skill as an Antecedent of the Within-Team Processes of Shared Leadership

Political skill is defined as “the ability to effectively understand others at work, and to use such knowledge to influence others to act in ways that enhance one’s personal and/or organizational objectives” (Ahearn et al., 2004, p. 311). It has been described as a social effectiveness variable which allows individuals to manage their relationships effectively (Blickle et al., 2008). The concept consists of four distinct but interrelated behavioral dimensions (Ferris et al., 2005): *Social astuteness* refers to heightened awareness of social environments and relationships as understanding others’ motivations and intentions. *Networking ability* refers to the ability to construct important relationships within networks in order to assemble a wide and heterogeneous support network of people. *Interpersonal influence* is the ability to influence others by adapting one’s own behavior and change influence tactics to successfully achieve desired responses. *Apparent sincerity* describes that politically skilled persons are being seen by others as honest and trustworthy, so as to reduce the chance that ulterior motives of the influencer can be detected by the influenced (Ferris et al., 2005).

Political skill is associated with accurate assessments of both one’s own and others’ motivations and needs, adequate situational appraisals and responses, favorable evaluations by others, and positive contributions to team and organizational processes (Ferris et al., 2007; Munyon et al., 2015; Frieder and Basik, 2017). Politically skilled individuals are described to know how to use influence tactics and strategies to evoke a favorable impression of themselves in their counterparts, which in turn leads to desired reactions and outcomes (*cf.* Ferris et al., 2007). Others “tend to view politically skilled individuals as trustworthy, credible, accountable and likable” (Ferris et al., 2007, p. 307). Moreover, they are said “to attract and inspire others” (Ferris et al., 2007, p. 307) and political skill is positively associated with leadership effectiveness (Kimura, 2015). Based on these and similar findings, political skill has repeatedly been suggested as a predictor of shared leadership (Ferris et al., 2009, 2012; Russell et al., 2016; Xu et al., 2019) and there is first evidence that political skill enhances individuals’ likelihood of emerging as informal leaders (Munyon et al., 2015).

In line with this earlier research, we propose a positive relationship between individual team members’ political skill and their propensity to be relied on for leadership by their fellow team members (i.e., taking the leader role). Furthermore, we extend this earlier research by suggesting a positive relationship between team members’ political skill and their propensity to rely on fellow team members for leadership (i.e., taking the follower role). In the following, we draw on research on ILTs and IFTs (see Lord et al., 2020) to substantiate these hypotheses.

We argue that due to their social astuteness, politically skilled individuals have a superior understanding of the social relationships within their team. They understand what their fellow team members expect from prototypical leaders, respectively followers. Moreover, they correctly assess in which role they gain the most favorable outcomes for their team in a given situation. In combination with their ability for interpersonal influence, politically skilled team members are then able to adapt their own behavior to fit either into the prototypical leader or follower role within a given situation. In other words, they either gain others’ reliance on their leadership or are recognized as relying on others for leadership.

A similar argument is proposed by Wihler et al. (2017), who describe the social astuteness and interpersonal influence dimensions of political skill in terms of opportunity recognition and capitalization, i.e., the capability of facilitating favorable outcomes through appropriate action. Also McAllister et al. (2018) stress that political skill enables individuals to recognize opportunities for social influence through their social astuteness and networking abilities. In the team setting, politically skilled team members are well equipped to understand whether they can best achieve the team’s goals in offering leadership or engaging in follower behavior and will prototypically engage in either of those. In addition, their apparent sincerity helps them to gain credibility in either role while their networking ability grants them support from many of their fellow team members. For example, Blickle et al. (2011) support the positive relationship between individuals’ political skill and their reputation building in a longitudinal study, arguing that politically skilled individuals are in the best position to address various expectations within their role.

To conclude, we argue that political skill enables individual team members to appropriately analyze social situations within their team with the aim of understanding whether they can best achieve their team’s goals by either offering leadership or following the lead of others in the respective situation. Moreover, we suggest that team members high on political skill are able to engage in one or the other role in a prototypical way. We therefore propose:

*H1a*: Team members’ political skill is positively related to their propensity of being relied on for leadership by their fellow team members.

*H1b*: Team members’ political skill is positively related to their propensity of relying on other team members for leadership.

### The Moderating Role of Empowering Formal Leadership

Most organizations decentralizing their decision-making structures do not entirely abolish formal leadership authority in teams but rather opt for a combination of formal and shared leadership (Lee and Edmondson, 2017). In the following, we draw on the concepts of ILTs and IFTs to elaborate on how empowering formal leadership facilitates the within-team processes of shared leadership.

Empowering leadership is defined as “leader behavior directed at individuals or teams that involves delegating authority to employees, promoting their self-directed and autonomous decision making, coaching, sharing information, and asking for input” (Sharma and Kirkman, 2015; Lee et al., 2018, p. 2). As such, empowering leadership is often framed as the opposite of directive leadership, in which most power rests with the formal leader (e.g., Martin et al., 2013). Empowering leadership focuses on promoting employees’ self-management and removing their constraints of powerlessness. According to Ahearne et al. (2005), empowering leaders enhance the meaningfulness of work for their employees, foster their participation in decision making, express confidence regarding employee performance, and provide autonomy from bureaucratic constraints.

In line with earlier theorizing and evidence (Pearce and Sims, 2000; Carson et al., 2007; Hoch, 2013; Fausing et al., 2015), we suggest that empowering formal leadership fulfills two important functions with regard to facilitating shared leadership in teams. First, by delegating power to team members and supporting them to use this power for meaningful team objectives, empowering leaders establish a promotive context in which the team members feel encouraged to leverage their qualities for the goals of the team. Second, empowering leaders provide a positive role-model of combining their own (formal) leader role with relying on other team members for leadership (i.e., taking the follower role). Thus, they serve as a prototype for being both a leader and a follower. The positive impact of the formal leaders’ role-model on creating a context for followers to engage in similar behaviors has been empirically supported (Sumpter et al., 2017).

We argue that politically skilled individuals with their ability to interpret social situations accurately (i.e., social astuteness), their ability to connect well with others (i.e., networking ability) and to influence others (i.e., interpersonal influence) in a convincing way (i.e., apparent sincerity) are in a privileged position to take advantage of this promotive context created and the prototypical role-model provided by the empowering formal leader. We suggest that empowering formal leadership provides the permission, space, and guidance for politically skilled individuals to identify (Adriasola and Lord, 2020) and build a favorable reputation for themselves (Ferris et al., 2007) as being both a prototypical leader and follower in the team. Thus, empowering formal leadership facilitates both the processes in which politically skilled team members enhance their propensity to be relied on for leadership and to rely on leadership by their fellow team members.

In particular, by fostering participation in decision making and thus sharing power with the team, the empowering formal leader communicates and provides a role-model to team members that shared leadership is required and supported, hence encouraging them to use their political skill to adequately engage in either the leader or the follower role in a given situation. By expressing confidence in their high performance and enhancing the meaningfulness of work, the empowering formal leader explicitly encourages team members to use their political skill for the common goals of the team, which can mean either to take the lead or to follow other team members. In a similar vein, empowering formal leaders provide autonomy from bureaucratic constraints, thereby allowing the full potential of the positive relationship between political skill and each of the within-team processes of shared leadership to unfold.

In sum, our reasoning leads to the following hypotheses:

*H2a*: Empowering leadership by the formal leader moderates the positive relationship between individuals’ political skill and their propensity to be relied on for leadership by their team members. Under high levels of empowering leadership, the relationship is stronger than under low levels of empowering leadership.

*H2b*: Empowering leadership by the formal leader moderates the positive relationship between individuals’ political skill and their propensity to rely on other team members for leadership. Under high levels of empowering leadership, the relationship is stronger than under low levels of empowering leadership.

## Materials and Methods

### Participants and Procedure

We invited all 37 members of a research and development (R&D) department at a medium-sized German manufacturing company to take part in our study. We received 29 responses (78.3% response rate), including one manager (at the highest level), three middle managers as formal leaders, and 25 employees without a formal leadership role. The respondents in the department were mostly male (93.0%), on average 48.18 years old (SD = 10.68), had 17.55 (SD = 11.05) years of tenure and showed high levels of education, with 89.0% of them holding at least a bachelor’s degree. In our study, we consider the department as one team, because the department represents a primary work system (Trist, 1981) with a common goal (i.e., the development of large industrial machines) and high levels of interdependence between its members. Thus, our analysis focused on the relationships between all members (including the manager and the three formal leaders) of the department, while we controlled for the presence of sub-structures in this team (see section “Controls” below).^1^ It has to be noted that in social network analysis, the relevant units for the network-related analyses are the ties that exist in the network, which need to be distinguished from the number of respondents (i.e., the 29 members of the R&D department) between which these ties are established (see section “Measures”).

For our data collection, participants received an email-invitation from the research team, asking them to fill in an online questionnaire by using an individual access code to ensure data protection. Participants were informed that their participation was voluntary and were ensured anonymity and data protection in the final analysis. To achieve this, we recoded all participants’ names into anonymous numbers and separated the files containing the names from their answers to prevent any matching. After the initial invitation, a first reminder was sent out 2 weeks later with a second reminder 1 week after that.

### Measures

#### Shared Leadership

For the shared leadership network, we collected ratings of each team member by each other team member (including the manager and the three formal leaders; Marsden, 1990). According to prior research (Carson et al., 2007; Chrobot-Mason et al., 2016), we applied the one-item measure “How much do you rely on this individual for leadership?”^2^ on a 7-point Likert scale (ranging from 1—*not at all* to 7—*very much*). Later, we dichotomized these leadership ratings to meet the technical requirement (Robins and Lusher, 2013) of using binary network relationships in our analysis (see Casciaro and Lobo, 2008; Chrobot-Mason et al., 2016). Specifically, we considered values equal to or greater than five (i.e., above the neutral point of four) as a leadership tie between participants, indicating that one team member had rated another team member as a source of leadership influence.^3^ With this dichotomization, we removed weaker ties from our network and focused solely on those ties that were perceived by participants to be fairly strong and influential (Chrobot-Mason et al., 2016; White et al., 2016).

#### Political Skill

Participants rated their own political skill using the 18-item measure by Ferris et al. (2005) on a 7-point Likert scale (1—*does not apply* to 7—*applies completely*). This instrument is the most widely established measure of political skill and has demonstrated very good construct validity (Ferris et al., 2005). It captures the four dimensions of the construct: social astuteness (e.g., “I am particularly good at sensing the motivations and hidden agendas of others”), networking ability (e.g., “At work, I know a lot of important people and am well connected.”), interpersonal influence (e.g., “I am good at getting people to like me”), and apparent sincerity (e.g., “It is important that people believe I am sincere in what I say and do”). Cronbach’s alpha for this scale was 0.89, while omega total was 0.95 (McNeish, 2018).

#### Empowering Formal Leadership

We asked participants to rate their individual perception of the extent to which their formal leader showed empowering leadership behaviors to them using the 10-item scale by Ahearne et al. (2005) with a 7-point Likert scale (1—*completely disagree* to 7—*completely agree*). This scale has shown very good psychometric properties (e.g., Zhang and Bartol, 2010; Cheong et al., 2016) and measures leaders’ enhancing the meaningfulness of work (e.g., “My manager helps me understand the importance of my work to the overall effectiveness of the company”), fostering participation in decision making (e.g., “My manager often consults me on strategic decisions”), expressing confidence in high performance (e.g., “My manager believes that I can handle demanding tasks”), and providing autonomy from bureaucratic constraints (e.g., “My manager makes it more efficient for me to do my job by keeping the rules and regulations simple”). By using an individual-level referent and asking about behaviors of the formal supervisor, this scale focuses on the relationship between the individual team members and their respective leader to obtain individual ratings of empowering leadership perceptions. Cronbach’s alpha for this scale was 0.89, while omega total was 0.98.^4^

#### Control Variables

First, we controlled for team members’ age as older team members might be more likely to be perceived as leaders based on their chronological age (Buengeler et al., 2016).

Second, we controlled for the sub-structure of the team by accounting for team members’ assignment to the same formal leader, which may increase the likelihood of tie formation. As we have outlined above, team members were strongly required to interact with one another across the entire team to achieve common goals; yet, interactions might be more likely between subordinates of the same formal leader due to closer proximity. In addition, leadership ratings could also potentially be influenced when team members had the same formal leader (Rivera et al., 2010).

Third, we controlled for formal hierarchical status, since formal leaders are often seen as significant sources of influence due to their formal position and therefore receive higher numbers of nominations (Chrobot-Mason et al., 2016).

Fourth, we accounted for the role of team and organizational identification. Prior research has shown both sources of identification to impact within-team processes of shared leadership, as team members with high identification are perceived to embody the goals and values of the team/organization and thereby are more likely be a source of leadership (Chrobot-Mason et al., 2016). We measured team identification using the 12-item scale by Henry et al. (1999) on a 7-point Likert scale (1—*completely disagree* to 7—*completely agree*) with questions like “all members need to contribute to achieve the group’s goals.” Cronbach’s alpha for this scale was 0.80; omega total was 0.85. Additionally, we used the one-item measure by Bergami and Bagozzi (2000) in which team members used a graphical representation of the overlap between their self-identity with the organizational identity. The individual-level correlations for all collected variables from the participants are presented in Table 1.

**Table 1:** Correlations, means, and standard deviations of the study variables.

Measure	*M*	SD	1	2	3	4	5	6	7	8	9	10
1 Age	48.18	10.74	–									
2 Political skill (PS)	4.71	0.78	0.28	–								
3 PS networking	4.15	1.11	0.27	0.89^***^	–							
4 PS interpersonal influence	5.09	0.81	0.28	0.33	0.11	–						
5 PS apparent sincerity	5.74	1.21	0.16	0.73^***^	0.47^**^	0.06	–					
6 PS astuteness	4.55	0.91	0.17	0.90^***^	0.71^***^	0.22	0.70^***^	–				
7 Team identification	5.74	0.71	0.12	0.07	0.11	0.25	−0.21	0.05	–			
8 Organizational identification	5.44	1.29	0.00	0.39^*^	0.41^*^	−0.17	0.30	0.44^*^	0.40^*^	–		
9 Empowering leadership	5.14	1.06	0.14	−0.02	0.03	−0.20	−0.0	0.03	0.53^**^	0.50^**^	–	
10 In-degree centrality	10.68	8.23	0.39^*^	0.48^*^	0.55^**^	−0.04	0.31	0.38^*^	0.33	0.53^**^	0.58^**^	–
11 Out-degree centrality	12.82	8.13	0.20	0.31	0.30	−0.16	0.23	0.36	−0.09	0.16	0.06	0.11

*** *p < 0.001;*

** *p < 0.01;*

* *p < 0.05*.

### The Shared Leadership Network

As indicated above, in social network analysis, the relevant units for the network-related analyses are the ties established between the members in the network. Thus, in our study, we obtained a total of 305 leadership and followership ties between the 29 respondents.^5^ A leadership tie between two members is established if one team member relies on the other for leader- or followership. The shared leadership network for our analysis was constructed by modeling all received ties (i.e., being relied on by others) and sent ties (i.e., relying on others for leadership). Our focus on one department (i.e., one network) and the size of our network are comparable to established research (Chrobot-Mason et al., 2016; Vega Yon et al., 2021).

Table 2 shows the descriptive statistics of the shared leadership network. With a sample of 29 participants (i.e., nodes), this network consisted of 305 realized leadership ties between the nodes with an overall network density of 0.38. Density is the measure of realized ties in relation to the number of possible ties (Carson et al., 2007), which indicates that in this network 38% of the possible leadership ties between participants existed. Compared to the average density value of 0.20 (SD = 0.40) found in the meta-analysis by D'Innocenzo et al. (2016), we consider this a medium to high level of shared leadership. In-degree centralization, i.e., the sum of differences in centrality between the most central members in a network and all others, was 0.61. This indicates that leadership tended to be somewhat centralized in relatively few actors. The reader may note that centralization and density measures are not proportional to one another, i.e., networks with low centralization do not necessarily have a high density or vice versa (D'Innocenzo et al., 2016). Taken together, the density and centralization measures indicated that we found medium to high levels of shared leadership in the observed network with several central actors having strong leadership influence over other team members.

**Table 2:** Descriptive network statistics of the leadership network.

Network statistic	Value
Density	0.38
Average degree	10.52
In-Degree centralization	0.61
Reciprocity	0.46
Average geodesic distance	1.3
Number of nodes	29
Number of ties	305
Maximum number of ties	812

**Figure 2:**
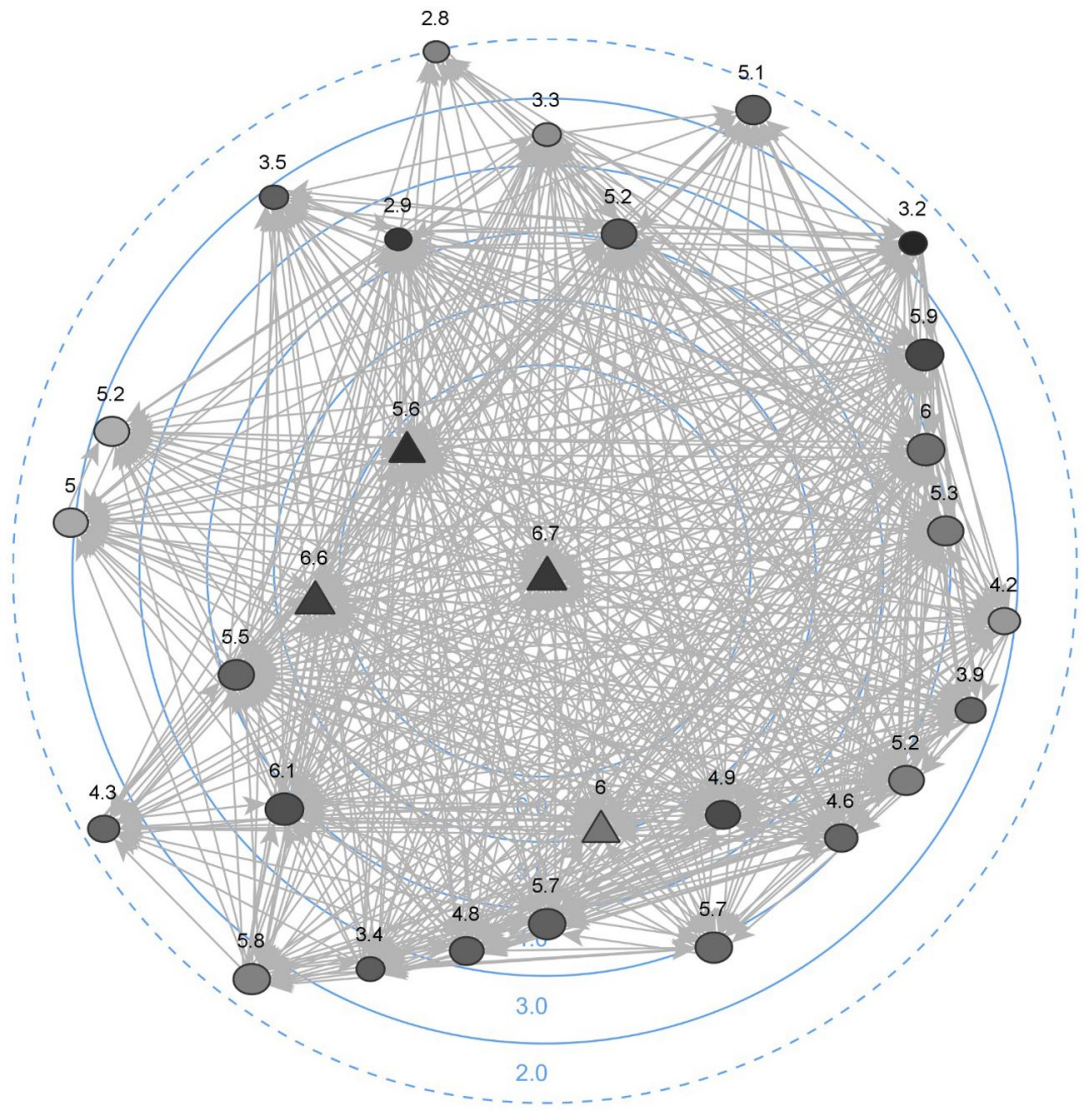
Visualization of the shared leadership network. Circles are team members and triangles represent formal team leaders. Darker coloration indicates higher levels of political skill, while numbers indicate perceived empowering leadership (1 = low, 7 = high). Position towards the center indicates more incoming leadership nominations.

A visual inspection of Figure 2 confirms this description. Figure 2 indicates that the team members (here, the circles), with an average of 8.32 (SD = 1.19) leadership nominations, did indeed rely on one another for leadership. At the same time, the team manager and the middle managers (here, the triangles) had significantly more leadership influence than other team members (*df* = 27, *t* = −5.58, *p* < 0.001), with an average of 25.5 (SD = 1.55) leadership nominations. Thus, we found evidence for both shared and formal leadership within this network.

### Analysis: Social Network Analysis Using Exponential Random Graph Models

**Figure 3:**
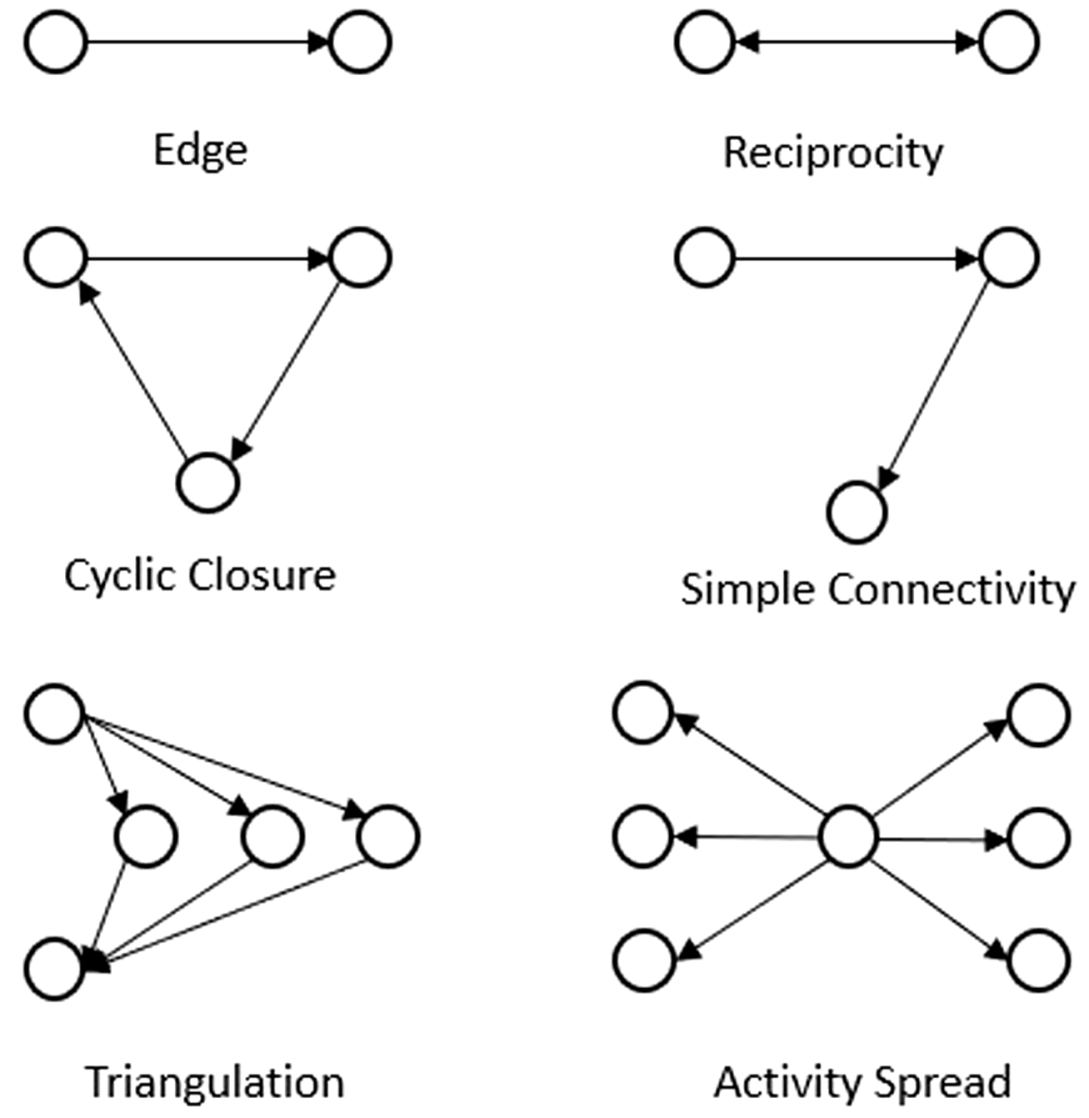
Selected subgraphs in social networks included in the analysis.

Due to the interdependent nature of relationships in networks, the common assumption of independence between observations in many standard analytical methods (e.g., regression analysis) is not met. To test our hypotheses, we therefore draw on the social network methodology, which explicitly assumes that actors and observations in networks are dependent on one another (Kilduff and Krackhardt, 2008; Snijders, 2011; Robins and Lusher, 2013). Specifically, we used exponential random graph models (ERGMs) applying the STATNET package (Handcock et al., 2016) in R (R Development Core Team, 2008). ERGMs are probability models developed especially for networks and allow to analyze the occurrence of (leadership) ties dependent on several observed variables (Robins and Lusher, 2013). Networks (see Figure 2 for our leadership network) consist of nodes (i.e., actors) and ties (i.e., relationships between actors). Networks have certain inherent attributes (i.e., network attributes) that are based on general human tendencies such as reciprocity (i.e., the tendency to reciprocate social behavior toward others) and homophily (i.e., the tendency to seek out others that are similar to oneself; Rivera et al., 2010). A typical example of these network attributes is the number of triangle relationships that are observable within the network, which reflects a higher likelihood of forming relationships with “friends of a friend” (e.g., if A is a friend of B and B is a friend of C, C has a higher chance of also being a friend of A; for an illustration of this and other examples see Figure 3). ERGMs simulate and compare possible ways in which the tie constellation in the observed network might have emerged based on the estimation and weighing of these network attributes together with attributes of the actors (i.e., non-network attributes; Robins and Lusher, 2013).

In our case, we used the ERGMs method to estimate the relative weight of the non-network attributes (i.e., political skill, perceptions of empowering leadership, and control variables) of our 29 actors (i.e., nodes) and specific network attributes (e.g., reciprocity and triangle formations) for the formation of our observed network with its 305 ties (i.e., units of analysis). Thus, the model included the actors’ attributes of interest (i.e., political skill and perceptions of empowering leadership) while at the same time accounting for the influence of the network attributes and controlling for a range of non-network attributes (i.e., our control variables as specified above). Furthermore, the ERGMs method allowed to differentiate between sender effects (i.e., the effect of the respective attributes on relying on others for leadership) and receiver effects (i.e., the effects of the respective attributes on being relied on for leadership) which permitted us to test our hypotheses (Chrobot-Mason et al., 2016).

#### Network Attributes

Following standard procedures, we included the following network attributes to realistically model the network relationships in our shared leadership network (Lusher and Robins, 2013). First, the human tendency to reciprocate social behavior (Rivera et al., 2010). Drawing on the granting and taking perspective of leadership (DeRue and Ashford, 2010) and the reciprocal nature of shared leadership in particular (Pearce and Conger, 2003), we assumed that leadership behavior may also follow the tendency to reciprocate. We assured that our estimation of the effect of our main attributes is robust by controlling for the effects of these naturally occurring reciprocal tendencies. Second, we also accounted for naturally occurring open triangular relationships by including the so-called simple connectivity (“two-path”). Simple connectivity models the tendency of formation of “open” triangles, in which A has a relationship with B and B another one with C that is itself not connected to A. Third, we similarly accounted for naturally occurring closed triangular relationships by including a cyclic closure term. Cyclic closure measures “closed” triangles in which the relationship tie from C does relate back to A, closing the relationship triangle (Czarna et al., 2016). Fourth, we included the geometrically weighted edgewise shared partner distribution (triangulation). This captures higher order triangular constellations in the network, meaning the tendency to form closed groups by making connections to “friends of friends.” Thereby, several other actors are considered as potential intermediates between two actors (A and B). Fifth, we also accounted for the geometrically weighted out-degree distribution (activity spread) to capture the activity distribution of the nodes in the network, i.e., to capture actors that have a very high tendency to form ties with others (Hunter et al., 2008; Czarna et al., 2016). The last two constructs embody self-organizing higher order network controls and are recommended for inclusion in order to achieve a more realistic model (Hunter et al., 2008).

#### Non-network Attributes

As for non-network variables, we added the abovementioned control variables: age, sub-team structure, formal leadership status, and team and organizational identification. To more specifically model the influence of identification, we entered a sender effect and a receiver effect for both team and organizational identification. This procedure allowed us to examine specifically whether identification would increase the likelihood to be relied on as a leader (i.e., receiver effects) and/or the likelihood to rely on others for leaders (i.e., sender effect), since both effects can occur independently from one another. A positive sender effect indicates that higher levels of the variable will increase the likelihood of relying on others for leadership, while a positive receiver effect indicates that higher levels of the attribute in question increase the likelihood of being relied on for leadership.

#### Main Effects and Interaction Effect

For each main variable (i.e., political skill and empowering leadership) in our model and their interaction, we included both a sender and receiver effect. To test Hypotheses 1a and 1b, we added the direct effect of political skill as both sender and receiver effects to the model. To test Hypotheses 2a and 2b, we added the level of empowering formal leadership as perceived by each individual team member as a direct effect together with the interaction term of political skill and empowering leadership as both sender and receiver effects to the model. For the interaction term, we standardized political skill and empowering leadership, calculated the interaction term, entered it into the model, and analyzed it using the 305 ties as units of analysis.

## Results

To test our hypotheses, we specified three models. In our baseline model (Model 0), we included the network attributes as well as our control variables. We then added the main effect of political skill in Model 1, followed by the main effect of empowering leadership and the interaction between political skill and empowering leadership in Model 2. The results for each model are explained below and all models are summarized in Table 3.

**Table 3:** Maximum likelihood estimates of ERGMS for leadership ties.

		Model 0			Model 1			Model 2	
Effect									
	Estimate	*SE*	*p*	Estimate	*SE*	*p*	Estimate	*SE*	*p*
**Endogenous network controls**									
Edges	−4.09	1.69	0.015	−8.57	2.03	<0.001	1.45	2.16	0.502
Reciprocity	0.16	0.28	0.562	0.19	0.28	0.509	0.26	0.30	0.394
Simple connectivity	−0.27	0.03	<0.001	−0.28	0.03	<0.001	−0.28	0.03	<0.001
Cyclic closure	0.36	0.08	<0.001	0.34	0.08	<0.001	0.34	0.07	<0.001
Triangulation	−0.21	0.37	0.572	−0.19	0.37	0.597	−0.14	0.36	0.691
Activity spread	−3.75	2.44	0.124	−3.30	2.64		−3.58	2.38	0.133
**Exogenous network controls**									
Age	0.04	0.01	<0.001	0.04	0.01	<0001	0.03	0.008	<0.001
Team structure	0.94	0.18	<0.001	0.89	0.18	<0001	0.58	0.19	<0.001
Formal status: Middle vs. team manager	2.34	0.34	<0.001	2.15	0.34	<0.001	0.91	0.73	0.213
Formal status: Member vs. team manager	0.09	0.47	0.857	−0.42	0.51	0.399	−2.06	0.85	0.015
Team identification: Sender	−0.35	0.13	<0.001	−0.26	0.13	0.053	−0.45	0.74	0.627
Team identification: Receiver	−0.23	0.14	0.096	−0.16	0.14	0.243	−0.44	0.15	0.002
Organizational identification: Sender	0.48	0.10	<0.001	0.43	0.10	<0.001	0.32	0.15	<0.001
Organizational identification: Receiver	0.65	0.09	<0.001	0.60	0.10	<0.001	0.39	0.10	<0.001
**Main effects**									
Political skill: Sender				0.48	0.14	<0.001	0.85	0.17	<0.001
Political skill: Receiver				0.57	0.13	<0.001	1.02	0.16	<0.001
Empowering leadership: Sender							0.45	0.14	<0.001
Empowering leadership: Receiver							0.56	0.13	<0.001
**Interaction effect**									
Empowering leadership × political skill: Sender							0.24	0.12	0.049
Empowering leadership × political skill: Receiver							0.39	0.13	<0.001

The interaction “Empowering leadership × political skill: Sender” was not significant in a no-controls model. This finding can thus not be rigorously supported by our analysis and needs to be interpreted with caution.

First, considering the baseline model (Model 0), we found that reciprocity did not occur in our network, i.e., leadership is not reciprocated directly between team members (estimate = 0.19, *p* = 0.509). However, we found that both simple connectivity and cyclic closure had a significant effect. The negative effect of simple connectivity (estimate = −0.28, *p* < 0.001) and the positive effect of cyclic closure (estimate = 0.34, *p* < 0.001) indicate that leadership is less likely to occur in open triangle relationships (A- > B- > C- > D) and more likely to occur in closed triangles (A- > B- > C- > A). This seems to indicate that there is a general tendency within our network to form close leadership relationships in which leadership is exchanged not directly, but *via* intermediaries. With regard to the control variables, we found a matching effect for the team structure (estimate = 0.89, *p* < 0.001), indicating that leadership ties are more likely to form between employees working under the same formal leader. We also found a significant effect for age (estimate = 0.04, *p* < 0.001), indicating that older team members have a slightly higher likelihood to form leadership ties with others compared to younger team members. Lastly, we found a significant negative effect for team identification and a significant positive effect for organizational identification. For these variables, we included both a sender effect for relying on others for leadership and a receiver effect for relying on others for leadership. For team identification, the likelihood of sending ties (estimate = −0.45, *p* = 0.002) and receiving ties (estimate = −0.44, *p* = 0.003) was reduced by higher levels of team identification. Contrariwise, organizational identification increased the likelihood of sending ties (estimate = 0.30, *p* = 0.003) and receiving ties (estimate = 0.36, *p* < 0.001). These two findings deviate somewhat from earlier research (Chrobot-Mason et al., 2016) but indicate that a strong identification with the team, compared to a stronger identification with the organization, does not enable team members to build, maintain, and utilize leadership relationships.

In Model 1, we entered political skill, including both sender and receiver effects to test Hypotheses 1a and 1b. We found that higher levels of political skill are significant predictors of both sending ties (estimate = 0.48, *p* < 0.001) and receiving ties (estimate = 0.57, *p* < 0.001), thus supporting both hypotheses.

In Model 2, in support of Hypotheses 2a and 2b, we found that the interaction of the individual team member’s political skill and the team members’ perception of their formal leader’s empowering leadership significantly increases the likelihood of both receiving ties (estimate = 0.39, *p* < 0.001) and sending ties (estimate = 0.24, *p* = 0.0496; see Table 3, Model 3).

To further test the robustness of our findings (Bernerth and Aguinis, 2016), we re-ran our analyses without control variables (i.e., age, sub-team structure, formal status, and team and organizational identification). Results revealed that all effects remained significant (*p* < 0.01), except for the interaction between political skill and empowering leadership on the probability of sending ties. Thus, although the choice of our control variables was based on sound theoretical considerations and followed recommendations to prevent omitted variable bias (Antonakis et al., 2010), the significant finding regarding Hypothesis 2b appears to be sensitive to our control variables and, hence, may be subject to statistical biases (Bernerth et al., 2018). Therefore, we concur with Sturman et al. (2022) that “if a hypothesis is framed in a way that describes a bivariate relationship, and if only the multivariate relationship is significant, the readers […] should consider that relationship more carefully.” Specifically, we caution readers that our theoretical assumption regarding the positive interactive effect of political skill and empowering leadership on the probability of relying on others for leadership (i.e., Hypothesis 2b) cannot be rigorously supported by our empirical analysis.

**Figure 4:**
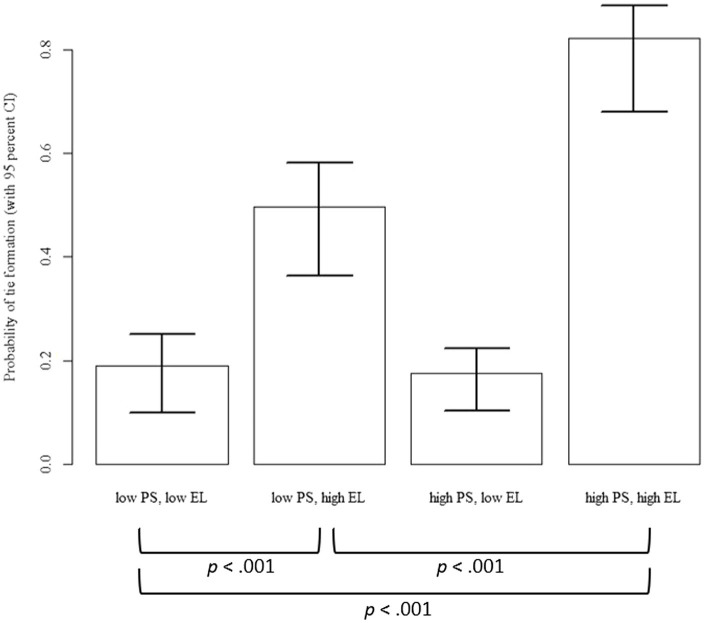
Interaction plot between political skill (PS) and empowering leadership (EL) for being relied on for leadership (i.e., receiver effects).

To further investigate the political skill-empowering leadership interaction for Hypothesis 2a, we used a micro-level analysis (Desmarais and Cranmer, 2012; Czarna et al., 2016) to generate an interaction plot (see Figure 4). We calculated combinations of individually perceived empowering leadership and political skill using the top quartile of both variables with the median tie formation probability using bootstrapping with 95% confidence intervals and 10,000 draws following the procedure outlined by Czarna et al. (2016). The resulting graph demonstrates the differences between combinations of political skill and individually perceived empowering leadership and their influence on the probability of receiver tie formation.

With regard to being seen as a leader, individuals with high levels of political skill and high perceived empowering leadership (i.e., high PS and high EL) showed the highest likelihood of receiving ties. We tested the difference between this and all other combinations of predictor and moderator with a Welch t-test (Czarna et al., 2016) and found all differences to be significant (all *p* < 0.001). The combination of low political skill and high empowering leadership (i.e., low PS and high EL) also showed an increased likelihood of receiving leadership ties, and this combination was also significantly different from all other combinations (all *p* < 0.001). The remaining two combinations with low empowering leadership and either high or low political skill (i.e., low PS, low EL; high PS, and low EL) had the lowest likelihood of receiving ties and did not differ significantly from each other (*p* = 0.966). Our results show that individuals with higher levels of political skill, compared to lower levels, were not more likely to be seen as sources of leadership unless they perceived high levels of empowering leadership from their formal leaders. This supports our assumption that empowering leaders enable and motivate their followers to use their political skill within the context of shared leadership.

We also inspected the interaction postulated in Hypothesis 2b, although we strongly caution readers that the significant interaction regarding this hypothesis appears to be sensitive to the control variables included and therefore does not receive rigorous support from our empirical analysis. Still, the pattern described in the following may be considered as a first tentative finding that might profit from further empirical exploration. Specifically, for relying on others for leadership (i.e., sending ties), individuals with high levels of political skill and perceived empowering leadership showed a much lesser degree of differentiation than for the received ties. More specifically, in the statistical analyses including control variables, we found no differences between combinations of a) low political skill and high empowering leadership (i.e., low PS and high EL), b) high political skill and low empowering leadership (i.e., high PS and low EL), and c) high political skill and high empowering leadership (i.e., high PS and high EL; for all *p* > 0.10). At the same time, we found that the combination of low political skill and low empowering leadership (i.e., low PS and low EL) had a significantly lower tie probability than the combinations of a) low political skill and high empowering leadership (i.e., low PS and high EL) and b) high political skill and high empowering leadership (i.e., high PS and high EL; *p* for all <0.10). Although these findings need to be considered with caution, our results here may be seen as tentative evidence that for relying on others for leadership, either one’s own political skill or one’s perception of empowering leadership from one’s formal leader might be sufficient to increase the likelihood of nominating others as sources of leadership.^6^

## Discussion

With the current study, we advance the literature on the emergence of shared leadership in teams. Specifically, we use social network analysis to investigate antecedents of the thus far under-explored within-team processes that constitute shared leadership. In our model and analysis, we focus on variables that increase both team members’ propensity to be relied on for leadership (i.e., the leader role) and their propensity to rely on others for leadership (i.e., the follower role). Drawing on research on ILTs and IFTs, which acknowledges the potential difficulty of moving from one role to the other (Lord et al., 2020), we identify individual team members’ political skill (Friedrich et al., 2009; Ferris et al., 2012) as a predictor for filling in each of these roles. Our findings support the importance of political skill as a social quality that fosters the emergence of shared leadership in teams. Further, we theorize that empowering leadership by the formal leader would provide an ideal context and prototypical role-model for team members to engage in both leader and follower roles. Our results partially support this hypothesis, as empowering formal leadership indeed strengthens the positive effect of team members’ political skill on their propensity to be relied on by others for leadership.

### Theoretical and Practical Implications

Our findings contribute to the extant theoretical understanding of shared leadership. Most of the existing literature investigates antecedents of shared leadership at the aggregate (i.e., team) level; our analysis extends this prior knowledge to the within-team processes that constitute shared leadership. In doing so, we go beyond prior research on shared and emergent leadership by setting our study at the intersection between the two literatures. In particular, we account for the fact that shared leadership requires both leader and follower roles (DeRue and Ashford, 2010), which raises the question of what drives team members’ engagement and acceptance of one another in each of these roles. This question has only scarcely been tackled in the extant literature. Our choice of political skill was informed by research on ILTs and IFTs (Lord et al., 2020). From this perspective, we looked for a variable that allows team members to engage in both, prototypical leader and follower behaviors. Our findings support political skill as an important variable that allows team members to navigate both sides of the within-team processes of shared leadership. Thus, our study contributes to the shared leadership literature by providing a deeper understanding of individuals’ contribution to the different requirements of shared leadership. Moreover, it adds to the emergent leadership literature, which to date has not considered social skills such as political abilities (Acton et al., 2019), but rather focused on individual abilities. Furthermore, our approach and findings contribute to the political skill literature by positioning the concept within the network leadership literature (Scott et al., 2018) and thus suggesting a new line of research beyond the impact of political skill on formal leaders’ emergence and effectiveness (Kimura, 2015).

With regard to the role of formal leadership, the within-team level of analysis offers a new perspective on the influence of empowering formal leadership on shared leadership. While extant research showed a direct influence of empowering formal leadership on shared leadership at the team level (Hoch, 2013; Jain and Jeppesen, 2014; Fausing et al., 2015), researchers have pointed out the necessity to expanded upon individual abilities by including contextual variables (Wellman et al., 2019; Hanna et al., 2021). By following this call, we provide a within-team perspective that allows for a more fine-grained analysis of the influence of empowering formal leadership, thereby considering it as a moderator of the relationship between individual team members’ political skill and the assumption of leader and follower roles. Thus, the shift in the level of analysis allows us to provide a refined explanation for the effectiveness of empowering formal leadership in a team context.

Empowering formal leadership moderates the relationship between political skill and being relied on for leadership. Our results suggest that political skill is clearly related to being relied on for leadership by other team members when empowering formal leadership is high, while it seems to have little impact when empowering formal leadership is low. This pattern suggests that empowering formal leadership facilitates an environment which makes it easy for politically skilled team members to be relied on by others for leadership, i.e., supports these team members in leading others by making use of their political skills. As for relying on others for leadership, we were not able to obtain robust empirical results that are consistent both with and without considering additional theoretically meaningful control variables. Therefore, the findings in our analysis can at best be considered as first tentative evidence that high levels of political skill might be more important to rely on others for leadership when empowering leadership is low. Overall, our findings indicate that political skill is an important team member quality, which allows the politically skilled individual to take advantage of empowering formal leadership in gaining their fellow team members’ reliance on their leadership influences.

Our pattern of findings on the joint influence of individual team members’ skills and empowering leadership also resonates with more general findings showing empowering leadership to facilitate intra-team coordination processes. For example, Carmeli et al. (2011) found that the empowering leadership by the CEO facilitates relevant team coordination processes (i.e., behavioral integration) in top management teams. Further, Oedzes et al. (2019) showed a dampening effect of empowering leadership on the negative relationship between informal hierarchies and team creativity. Our findings also add to reasoning brought forward by Wellman et al. (2019) who argue that just providing freedom via laissez-faire leadership does not encourage team members to take up the role of informal leaders. Rather, these authors propose that a motivational component in the sense of active role modeling is required to encourage leadership emergence. Our findings are in line with these arguments. However, these studies still focus on the team level. To fully understand the within-team processes of shared leadership—as considered in our study—and the impact of team members’ characteristics as well as formal leadership influences, further research is required which includes a variety of additional team member characteristics and formal leadership behaviors.

Our research is of high practical relevance as practitioners can learn from our study how to foster shared leadership in their organizations. First, based on our findings, they might be well-advised to select employees for their teams based on their political skill and/or to train them to fully develop their political skill. Ferris et al. (2000) have already shown how individuals’ political skill can be trained successfully. Second, our findings on the moderating role of empowering formal leadership give practitioners a better understanding of how formal leaders can at least partially enable shared leadership processes in their teams. Therefore, organizations should focus on including empowering leadership into their formal leadership trainings to facilitate shared leadership in the leaders’ teams. First evidence on the trainability of empowering leadership is already available, for example, from Lorinkova et al. (2013).

### Limitations and Recommendations for Future Research

As with all empirical research, our study is not without limitations that need to be considered when interpreting our results. First and foremost, we acknowledge that our study is based on a rather small and selective sample which naturally inhibits the generalizability of our findings. Specifically, our sample comprises members of only one department from one company. Although such a limited empirical setting is not uncommon in research based on social network analysis (Vega Yon et al., 2021) which requires rather complex data collection procedures (for a comparable research setting and sample, see, e.g., Chrobot-Mason et al., 2016), the external validity of our findings and their generalizability to other contexts (e.g., larger units in other functional areas or organizations in different industries) are likely to be limited (Shen et al., 2011). In addition to the selective setting and limited sample size, our subjects of analysis (i.e., the members of the R&D department) showed rather little variety in terms of demographic characteristics: The respondents in our study were mostly male, middle aged, and with high levels of education, which limits the generalizability of our results to other contexts with more diverse team compositions (e.g., Muethel et al., 2012). Nonetheless, our method based on social network analysis yielded 305 relationships for investigation that allow for a closer look at the micro-level interactions of actors in a given network. Still, given the undeniable limitations of our empirical setting, we strongly encourage future research to replicate our findings with larger and more heterogeneous samples from different units in a variety of organizations to warrant better generalizability of the findings. This may also include the investigation of more than one network (e.g., White et al., 2016).

Second, our research design is cross-sectional in nature, with all variables measured at the same point of time. Although we suggest that our model is based on sound theoretical reasoning, no causal claims can be made on the relationships identified in our analysis, and the possibility of reverse causality cannot entirely be ruled out (Antonakis et al., 2010). Thus, future research would strongly profit from more longitudinal research designs using several data collection points to analyze the development and potential temporal trajectories of shared leadership networks over time, in particular with regard to the role of political skill and empowering leadership (Czarna et al., 2016).

Third, we could not yield robust support for the assumption that empowering leadership facilitates politically skilled team members’ propensity to rely on others for leadership. Our robustness checks revealed that the significance of the interaction depends on additional control variables. The choice of these variables was guided by sound theoretical considerations and prior empirical evidence with the aim of preventing omitted variable bias and holding theoretically and empirically meaningful influence factors of leadership emergence constant (Antonakis et al., 2010). However, the pattern of a non-significant interaction in the no-controls model may also be the result of statistical biases, such as suppression effects (Bernerth et al., 2018). Therefore, future research would benefit from further exploring the interaction between political skill and empowering leadership in predicting the reliance on others for leadership to yield more robust empirical evidence, for example, in a larger sample with increased statistical power.

Fourth, our findings suggest that team members’ political skill facilitates their emergence in both the leader and the follower role in their team. Moreover, political skill has been shown to positively contribute to team performance (Ahearn et al., 2004; Lvina et al., 2018) and has been referred to as one of the most important competencies of a leader (Treadway et al., 2004). However, political skill is conceptualized as a neutral phenomenon, with its effects being determined by the underlying intentions (*cf.* Ahearn et al., 2004). Organizations might be worried that training their members in developing political skill will help them to promote their individual rather than the team’s and organization’s goals. Therefore, we suggest to include context variables into future research that foster a pro-team/organizational use of political skill (e.g., commitment to shared goals and incentives based on team success).

We understand our model as a first step toward a more comprehensive framework capturing antecedents of the within-team processes of shared leadership. We encourage future research to focus on variables that allow individual team members to engage in and be accepted in both the leader and the follower role, qualities that enable flexibly switching between the roles, as well as context factors that facilitate these processes. This recommendation is in line with the emergent leadership framework by Hanna et al. (2021), which suggests that leader (and follower) emergence is determined by a combination of team member qualities and team context variables. Researchers may identify these variables in further drawing on work from the ILT/IFT literature (Lord et al., 2020), work on leader and follower identity (DeRue and Ashford, 2010; DeRue et al., 2011), or recent insights on the paradoxical relationship between formal and shared leadership (Pearce et al., 2019).

## Data Availability Statement

The datasets presented in this article are not readily available because data were collected in cooperation with an industry partner that restricts any publication of the dataset due to data protection requirements. Requests to access the datasets should be directed to sebastian.tillmann@uni-konstanz.de

## Ethics Statement

Ethical review and approval were not required for the study on human participants in accordance with the local legislation and institutional requirements. The patients/participants provided their written informed consent to participate in this study.

## Author Contributions

All authors listed have made a substantial, direct, and intellectual contribution to the work and approved it for publication.

## Funding

This article received generous funding for open access publication by the Bundeswehr University Munich.

## Conflict of Interest

The authors declare that the research was conducted in the absence of any commercial or financial relationships that could be construed as a potential conflict of interest.

## Publisher’s Note

All claims expressed in this article are solely those of the authors and do not necessarily represent those of their affiliated organizations, or those of the publisher, the editors and the reviewers. Any product that may be evaluated in this article, or claim that may be made by its manufacturer, is not guaranteed or endorsed by the publisher.

## Supplementary Material

The Supplementary Material for this article can be found online at: https://www.frontiersin.org/articles/10.3389/fpsyg.2022.866500/full#supplementary-material

## Footnotes

^1^In doing so, we follow seminal works conceptualizing leadership as a relational construct (for a recent review, see Kim et al., 2020), such as works on the vertical dyad linkage (e.g., Dansereau et al., 1975) and individualized leadership (e.g., Dansereau et al., 1995), postulating that individuals develop unique leadership relationships with their formal leaders. This relationship perspective is also mirrored in the empowering leadership literature which has conceptualized empowering leadership as a leadership style targeted at individuals (e.g., Sharma and Kirkman, 2015) and has provided evidence for individualized relationships between empowering leaders and followers (e.g., Zhang and Bartol, 2010; Li et al., 2017).

^2^All items for all measures were translated into German using back-translation procedures as outlined by Brislin (1986) when no validated German versions were available.

^3^As a robustness check for the dichotomization, we followed recommendations by Cranmer and Desmarais (2011) and alternatively conducted the analysis with a dichotomization at values greater than or equal to six, resulting in a less dense network. However, this alternative dichotomization did not impact our results.

^4^As in prior leadership research using a social network approach (e.g., Emery et al., 2011; Chrobot-Mason et al., 2016), the small sample-size-to-parameter ratio in our study prevented the possibility to conduct a confirmatory factor analysis to test the construct validity of our scales (Kline, 2005; Brown, 2006). Still, the focal variables in our study (i.e., political skill and empowering leadership) are widely established measures for which the construct validity has been proven in prior analyses (see, e.g., Ferris et al., 2005; Zhang and Bartol, 2010; Cheong et al., 2016).

^5^As we obtained 29 responses from the 37 members of the R&D team and social networks can be susceptible to missing ties, we conducted a comparative t-test on the number of leadership nominations for participants (*M* = 10.68, SD = 1.53) and non-participants (*M* = 8.37, SD = 2.10) that showed no significant difference in the number of nominations [t(35) = 0.74, *p* > 0.05], indicating that non-participants were not substantially isolated and therefore are likely missing at random.

^6^To further test the reliability of our analysis, we conducted a degeneracy and goodness-of-fit analysis. Results are available in the **Appendix** (see Supplementary Material).
