# Supplementary material for: When Do Team Members Share the Lead? A Social Network Analysis
Source: Front Psychol. 2022 Apr 25;13:866500. doi: 10.3389/fpsyg.2022.866500 (PMC9083072; doi:10.3389/fpsyg.2022.866500)
Supplement: Supplementary file 1 [file Presentation_1.pdf]

Appendix: Degeneracy and Goodness-of-Fit Analysis

To test the reliability of our analysis, we conducted a test of model degeneracy using the inbuilt function of the STATNET package (Handcock et al., 2016). This function tests whether models are poorly specified, as this would lead to unreliable estimations (Robins and Lusher, 2013). The non-significant *p*-values for either model (i.e., Model 0, $p = .910$; Model 1, $p = .343$; Model 2, $p = .36$; Model 3, $p = .432$) indicate that neither model is degenerate and therefore our results can be interpreted with confidence.

In a follow-up step, we investigated the goodness-of-fit by simulating a series of networks from our data and comparing Model 2 to the simulated networks. Ideally, Model 2 should capture a series of typical network parameters (i.e., in-degree, out-degree, edge-wise shared partners and minimum geodesic distance) in a similar way as the simulated networks in order to rule out a misspecified model (Lusher et al., 2013). A visual examination of the goodness-of-fit analysis in the figure below reveals that, while this is not the case for all variables, (i.e., we could not estimate the geodesic distances within 95% confidence intervals of the simulated networks), the overall model fit is satisfactory as most of the values for in-degree, out-degree and edge-wise shared partners fall within the 95% confidence interval. This result indicates that our model does not significantly deviate from a range of alternative networks simulated from the same data and therefore captures the network in a realistic way.

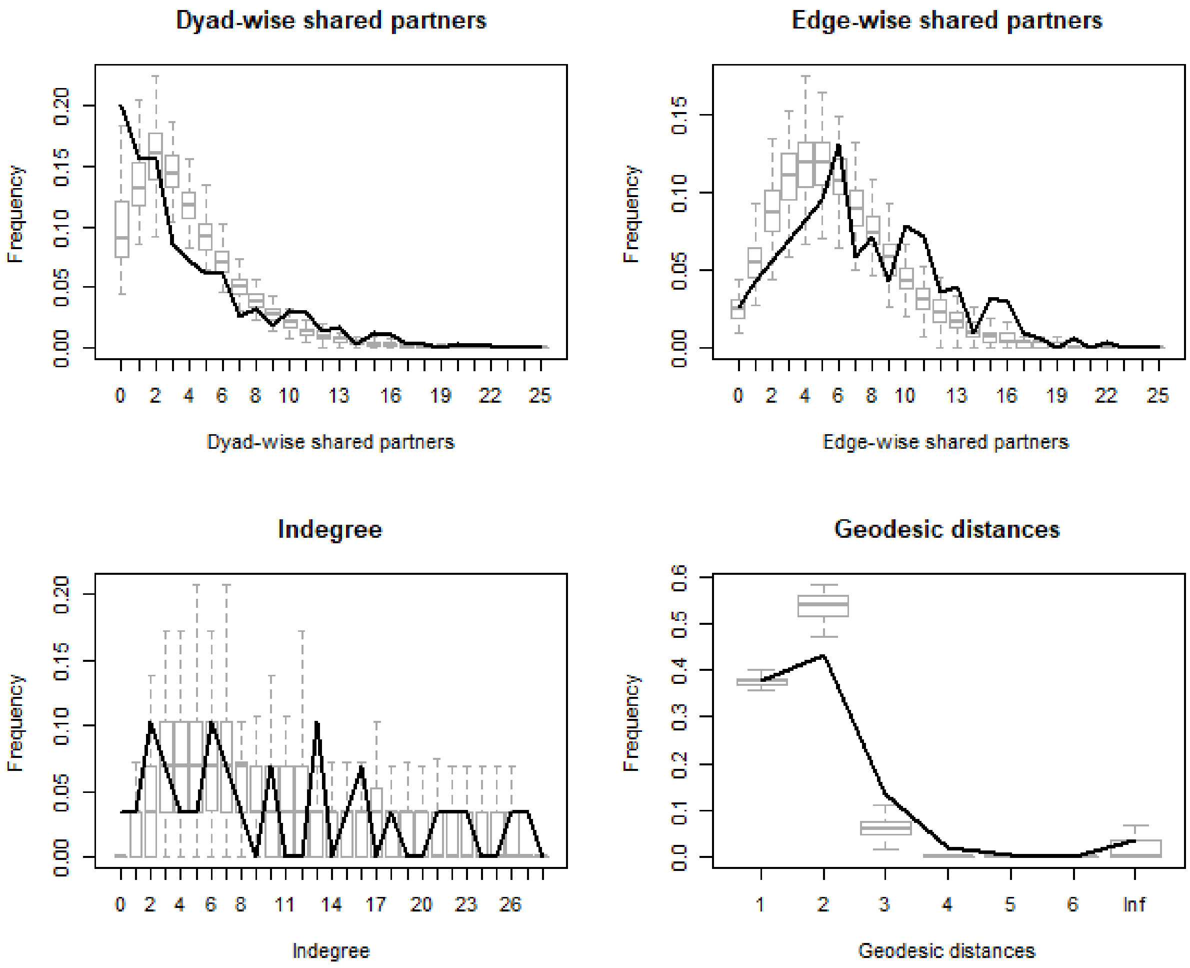

Figure: Visual representation of the goodness-of-fit assessment for the ERGMs.
